# A SMAD4‐modulated gene profile predicts disease‐free survival in stage II and III colorectal cancer

**DOI:** 10.1002/cnr2.1423

**Published:** 2021-06-10

**Authors:** Bryan C. Szeglin, Chao Wu, Michael R. Marco, Hyun Sung Park, Zeda Zhang, Bing Zhang, Julio Garcia‐Aguilar, R. Daniel Beauchamp, X. Steven Chen, J. Joshua Smith

**Affiliations:** ^1^ Colorectal Service, Department of Surgery Memorial Sloan Kettering Cancer Center New York USA; ^2^ Albert Einstein College of Medicine Bronx New York USA; ^3^ Human Oncology and Pathogenesis Program Memorial Sloan Kettering Cancer Center New York USA; ^4^ Weill Cornell Medical College New York USA; ^5^ Gerstner Graduate School of Biomedical Sciences Memorial Sloan Kettering Cancer Center New York USA; ^6^ Department of Molecular and Human Genetics and the Lester and Sue Smith Breast Center Baylor College of Medicine Houston Texas USA; ^7^ Section of Surgical Sciences Vanderbilt University Medical Center Nashville Tennessee USA; ^8^ Division of Biostatistics, Department of Public Health Sciences University of Miami Miller School of Medicine Miami Florida USA

**Keywords:** cancer biology, colorectal cancer, gene expression profile, SMAD4, tumor suppressor genes

## Abstract

**Background:**

Colorectal cancer is the second‐leading cause of cancer‐related mortality in the United States and a leading cause of cancer‐related mortality worldwide. Loss of SMAD4, a critical tumor suppressor and the central node of the transforming growth factor‐beta superfamily, is associated with worse outcomes for colorectal cancer patients; however, it is unknown whether an RNA‐based profile associated with SMAD4 expression could be used to better identify high‐risk colorectal cancer patients.

**Aim:**

Identify a gene expression‐based SMAD4‐modulated profile and test its association with patient outcome.

**Methods and results:**

Using a discovery dataset of 250 colorectal cancer patients, we analyzed expression of BMP/Wnt target genes for association with SMAD4 expression. Promoters of the BMP/Wnt genes were interrogated for SMAD‐binding elements. Fifteen genes were implicated and three tested for modulation by SMAD4 in patient‐derived colorectal cancer tumoroids. Expression of the 15 genes was used for unsupervised hierarchical clustering of a training dataset and two resulting clusters modeled in a centroid model. This model was applied to an independent validation dataset of stage II and III patients. Disease‐free survival was analyzed by the Kaplan‐Meier method. *In vitro* analysis of three genes identified in the SMAD4‐modulated profile (JAG1, TCF7, and MYC) revealed modulation by SMAD4 consistent with the trend observed in the profile. In the training dataset (*n* = 553), the profile was not associated with outcome. However, among stage II and III patients (*n* = 461), distinct clusters were identified by unsupervised hierarchical clustering that were associated with disease‐free survival (*p* = .02, log‐rank test). The main model was applied to a validation dataset of stage II/III CRC patients (*n* = 257) which confirmed the association of clustering with disease‐free survival (*p* = .013, log‐rank test).

**Conclusions:**

A SMAD4‐modulated gene expression profile identified high‐risk stage II and III colorectal cancer patients, can predict disease‐free survival, and has prognostic potential for stage II and III colorectal cancer patients.

## 1 INTRODUCTION

Colorectal cancer (CRC) is the second‐leading cause of cancer‐related mortality in the United States^1^ and a leading cause of cancer‐related mortality worldwide.^2^ Accurate recurrence prognostication is challenging, especially in stage II and III CRC where some patients are cured by surgical intervention alone^3^ and higher survival rates are observed in stage IIIb patients compared to stage IIc patients.^4^ Unfortunately, pathologic features associated with high‐risk stage II CRC have limited predictive accuracy,^5^ as do molecular risk factors such as microsatellite instability status and loss of 18q.^6^ An alternative prognostic tool is needed to identify high‐risk stage II and III patients who might benefit from adjuvant chemotherapy after surgical resection.

One prognostic biomarker in CRC is the tumor suppressor SMAD4, the central node in the transforming growth factor‐beta (TGF‐β) superfamily.^7^ Loss of SMAD4 has been associated with worse outcomes in stage III CRC patients^8^, ^9^ and resistance to 5‐fluorouracil‐based therapy *in vivo* and *in vitro*.^10^, ^11^, ^12^, ^13^ TGF‐β pathway inactivation is observed in approximately 30%–60% of CRCs^7^ and experimental evidence suggests that this pathway inhibits adenoma to adenocarcinoma conversion.^14^ The Wnt pathway is known to interact with the TGF‐β pathway during embryological development of the central nervous system^15^ and we previously reported that *SMAD4* restoration reduces β‐catenin levels to suppress Wnt signaling, upregulating bone morphogenetic protein (BMP)‐specific transcriptional targets.^16^ One proposed mechanism is that SMAD4 suppresses Wnt signaling via repression of target genes and upregulation of the BMP arm of the TGF‐β superfamily pathway.

Although a recent meta‐analysis validated SMAD4 immunohistochemistry (IHC) as a prognostic tool,^17^ RNA‐based signatures have gained traction as quantifiable alternatives to IHC and are utilized in various cancers for both diagnosis and prognosis.^18^, ^19^, ^20^ One example is Oncotype DX^21^ (Genomic Health) which uses a 21‐gene signature to quantitatively predict distant recurrence of breast cancer. Genomic Health applied this technology to stage II and III CRC, but while validation studies accurately predicted relapse‐free survival, they failed to predict treatment response.^22^, ^23^ Additional gene expression signatures have provided important insights into CRC heterogeneity,^24^, ^25^ and include subtype signatures,^26^ stromal signatures,^27^, ^28^ a metastatic expression signature,^29^ and a Wnt‐related signature.^30^ However, a specific RNA‐based profile associated with a tumor suppressor has not been utilized to identify high‐risk CRC. Synthesizing our understanding of the biology of SMAD4 in both the BMP and Wnt pathways, and with the findings from our previous studies, we hypothesized that a SMAD4‐modulated gene profile could help identify patients with high‐risk CRC and worse disease‐free survival.

## 2 MATERIALS AND METHODS

### 2.1 Ethics approval and consent to participate

Use of human tumor tissues was approved by the respective Institutional Review Boards. Use of patient‐derived tissues and subsequent experiments at MSK were approved by the Institutional Review Board under an approved protocol (Dr. Smith ‐ PI). Patients also consented for tissue use and sequencing on a separate protocol.

### 2.2 BMP/Wnt target gene lists

Gene ontology and bioinformatics curation tools were used to generate BMP and Wnt target gene lists. The BMP target list was generated with the GO tool (geneontology.org) and the targets were validated by manual search and verification in PubMed (Table S1). The Wnt target list was generated by reviewing the genes listed on the website http://web.stanford.edu/~rnusse/pathways/targets.html and validating them by manual search and verification via a literature search in PubMed (Table S2). Only genes supported as targets by published, annotated sources were used. The genes were then matched to Affymetrix U133 Plus 2.0 Array probe identifiers (Tables S3 and S4). We then generated a combined BMP/Wnt target gene list by identifying genes common to both lists.

### 2.3 SMAD4‐modulated gene profile

**FIGURE 1:**
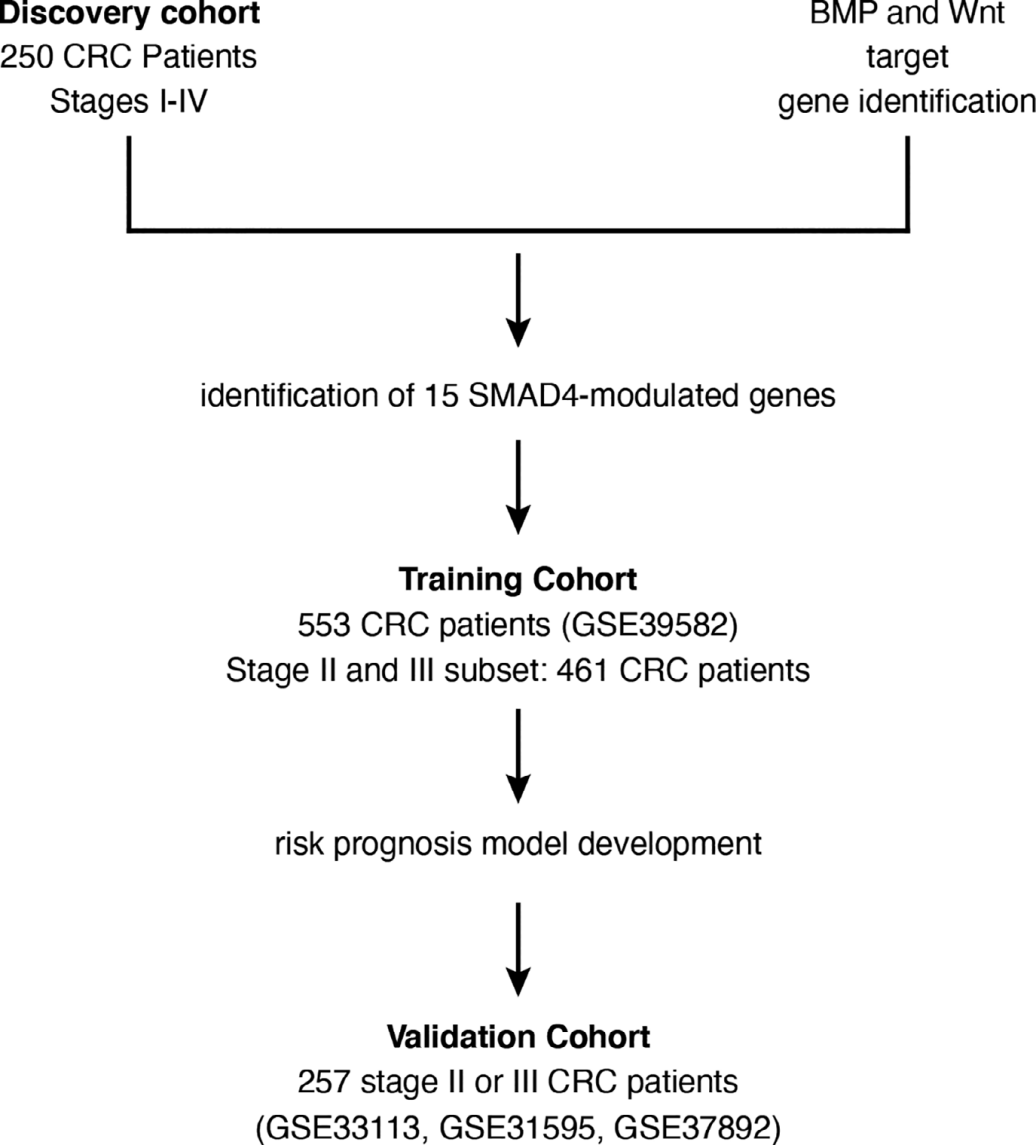
Study flow diagram. A discovery cohort of 250 colorectal cancer (CRC) patients from Vanderbilt University Medical Center (VUMC) and Moffit Cancer Center (MCC) was used to identify SMAD4‐modulated genes among known target genes of BMP and Wnt (see Figure 2 and Methods). An independent training cohort of 553 CRC patients was then used to identify a high‐risk group of stage II and III CRC patients. A risk prognosis model was developed and then validated in a separate, independent cohort of 257 stage II and III CRC patients

In a discovery set of tumors from 250 CRC patients from Vanderbilt University Medical Center (VUMC) and Moffit Cancer Center (MCC),^31^ SMAD4 expression levels (202527_s_at probe) were obtained from the data generated by the Affymetrix U133 Plus 2.0 Array platform (Figure 1). We selected 202527_s_at to represent SMAD4 based on its exon location and larger interquartile range. Note that the discovery dataset has been published to the Gene Expression Omnibus (GEO) database at GSE161158, and batch effect has been removed using *ComBat* function from Bioconductor package *sva*.^32^ We then used two approaches to find SMAD4‐modulated genes. First, we examined the correlation of expression levels of the genes in the BMP/Wnt combined list with SMAD4 expression levels to identify significant probes using Spearman correlation (False Discovery Rate adjusted *p*‐value < 0.01). Second, to identify BMP/Wnt target genes with SMAD4‐binding sites in the promotor region, we used the ExPlain Analysis System (Biobase) to retrieve promoter sequences and examine them for SMAD‐binding elements (SBEs).^33^ We then determined which probes and corresponding genes from the SBE analysis were correlated with SMAD4 expression. The probes and genes from these two approaches were combined and this was carried forward as the SMAD4‐modulated profile (Table 1).

**TABLE 1:** SMAD4‐modulated gene profile

Gene symbol (chromosome)	Gene name	Direction of correlation^a^	Number of SBEs
*DKK1* (10q)	Dickkopf WNT signaling pathway inhibitor 1	+	4
*VEGF‐A* (6p)	Vascular endothelial growth factor A	+	3
*WNT1* (12q)	Wingless‐type MMTV integration site family member 1	−	3
*TWIST1* (7p)	Twist family bHLH transcription factor 1	+	3
*SNAI1* (20q)	Snail family zinc finger 1	−	3
*SOX9* (17q)	SRY‐box 9	−	2
*DLL1* (6q)	Delta‐like 1 (human homolog of the drosophila Notch delta ligand)	+	2
*BTRC* (10q)	Beta‐transducin repeat containing E3 ubiquitin protein ligase	+	2
*ID2* (2p)	Inhibitor of DNA binding 2, dominant negative helix–loop–helix protein	+	2
*LEF1* (4q)	Lymphoid enhancer‐binding factor 1	+	2
*TCF7* (5q)	Transcription factor 7 (T‐cell specific, HMG‐box)	−	2
*MYC* (8q)	V‐Myc avian myelocytomatosis viral oncogene homolog (C‐Myc)	−	1
*JAG1* (20p)	Jagged 1 (ligand for Notch 1)	+	1
*TCF4* (18q)	Transcription factor 4 (TCF7L2)	+	1
*FN1* (2p)	Fibronectin 1	+	0

Abbreviation: SBE, SMAD‐binding element.

^a^ Indicates whether expression of the gene is positively or negatively correlated with *SMAD4* expression. Notably, 14 of the 15 genes have SBEs in their promoter sequences.

### 2.4 Validation of SMAD4 target genes *in vitro* using colorectal cancer tumoroids

#### 2.4.1 Colorectal cancer tumoroid cultures

Tumoroids were derived and maintained as described in Ganesh, Wu, O'Rourke, et al.^34^ Tumoroids were randomly tested for Mycoplasma contamination and those tested were negative.

#### 2.4.2 Crispr/Cas9‐mediated SMAD4 knockdown

A single‐guide RNA (sgRNA) sequence to target *SMAD4* was designed using the online tool developed by Boutros and colleagues^35^ (www.e-crisp.org/E-CRISP/) as described by Drost et al.^36^ The sequence was GATCAGGCCACCTCCAGAGA. The sgRNA oligomer was cloned into the LentiCRISPRv2 vector, and lentiviral particles were generated by transfecting HEK293T cells with the LentiCRISPRv2‐sgRNA construct, psPAX2, and VSV‐G.^37^ HEK293T cells (7.25 × 10^6^) were seeded in a 10 cm dish. The LentiCRISPRv2‐sgRNA construct (7.7 μg), psPAX2 (5.8 μg), and VSV‐G (3.9 μg) were delivered with Lipofectamine 2000 (Invitrogen) according to the manufacturer's protocol. Cells were grown overnight after transfection, and medium was replaced with the standard DMEM‐fetal bovine serum supplemented with GlutaMax and Pen‐Strep. At 2 days post‐transfection, the virus medium was filtered through a 0.45 μm filter and then concentrated using the PEG‐it Virus Precipitation Solution (System Biosciences) and the lentiviral particles were resuspended in 300 μl of infection medium (tumoroid culture medium plus 8 μg/ml hexadimethrine bromide [Polybrene; Sigma‐Aldrich] and 10 μM Y27632 [Sigma‐Aldrich]). After dissociation of the organoids (three 50 μl Matrigel discs per viral construct) with cell recovery solution (BD Biosciences), the cell clusters were resuspended in 10 μl of infection medium. The cell cluster suspension and viral suspension were combined in a 48‐well culture plate. The culture plate was centrifuged at 600 × *g* at room temperature for 60 min and subsequently incubated for 6 h in standard culture conditions (37°C with 5% CO_2_). The infection mixture was transferred to a 1.5 ml Eppendorf tube, the cells centrifuged to form a pellet, and the infection medium supernatant discarded. The cells were resuspended with 150 μl of Matrigel and divided into three wells of a 24‐well suspension plate. Matrigel was polymerized, 500 μl of infection medium without Polybrene was added, and the medium was replaced with culture medium 2 days after infection. The infected cells were selected by addition of puromycin (2 μg/ml) at 6 days.

### 2.5 Western blot

Cells were processed and lysed as previously described.^38^ The tumoroid samples were processed according to published methods.^39^ Equal amounts of protein were loaded in each lane of a sodium dodecyl sulfate 4%–12% polyacrylamide gel. Western blot analysis was performed by the standard method using the following primary antibodies: anti‐SMAD4 (ab40759; Abcam; 1:1000), anti‐TCF7 (2203S; Cell Signaling Technology; 1:1000), anti‐c‐Myc (9402S; Cell Signaling Technology; 1:1000), anti‐Jagged‐1 (sc‐8303; Santa Cruz Biotechnology; 1:200), and anti‐β‐actin (ab49900; Abcam; 1:10 000). Western blot images were analyzed, and bands were quantified using ImageJ software (version 1.50b; National Institutes of Health; https://imagej.nih.gov/ij/).

#### 2.5.1 Association between the SMAD4‐modulated gene profile and disease‐free survival

The potential clinical utility of the SMAD4‐modulated profile was evaluated by unsupervised hierarchical clustering in an independent training dataset of tumor samples from CRC patients, excluding stage 0 patients in this dataset (Figure 1, GSE39582; *n* = 553).^24^ Validation analysis was performed in three independent datasets of tumor samples from stage II/III CRC patients: GSE33113 (*n* = 90),^40^ GSE31595 (*n* = 37),^41^ and GSE37892 (*n* = 130)^42^ (Figure 1). The validation datasets were combined to optimize power (*n* = 257). All of the GSE datasets used for training and validation were downloaded from the Gene Expression Omnibus site (https://www.ncbi.nlm.nih.gov/geo/). All datasets were based on the Affymetrix U133 Plus 2.0 Array platform. Available characteristics for each patient dataset are summarized in Table 2. The Robust Multi‐Array Average algorithm in the Bioconductor *Affy* package was applied to pre‐process and normalize those Affymetrix microarray datasets. Association with available clinical variables was tested using Pearson's chi‐squared test. Disease‐free survival (DFS) was chosen as the clinical outcome of interest because it was available in all datasets and because it is a robust indicator of prognosis in CRC.^43^

**TABLE 2:** Patient demographics

	Discovery		Training		Validation	
	VUMC (*n* = 55)	MCC (*n* = 195)	GSE39582 (*n* = 562)	GSE33113 (*n* = 90)	GSE31595 (*n* = 37)	GSE37892 (*n* = 130)
	United States	United States	France	The Netherlands	Denmark	France
General						
Age, yrs, mean ± SD	62.3 ± 14.4	65.3 ± 12.9	66.8 ± 13.3	70.4 ± 13.0	74.1 ± 10.1	68.3 ± 12.7
Sex, male, *n* (%)	29 (52.7)	89 (45.6)	307 (54.6)	42 (46.7)	15 (40.5)	69 (53.1)
Race, *n* (%)			NA	NA	NA	NA
White	50 (90.9)	165 (84.2)				
Black	4 (7.3)	11 (5.6)				
Other or Unknown	1 (1.8)	19 (9.7)				
Stage, *n* (%)						
Stage I	4 (7.3)	29 (14.9)	33 (5.9)	0 (0.0)	0 (0.0)	0 (0.0)
Stage II	15 (27.3)	61 (31.3)	264 (47.0)	90 (100.0)	20 (54.1)	73 (56.2)
Stage III	19 (34.5)	63 (32.3)	205 (56.2)	0 (0.0)	17 (45.9)	57 (43.8)
Stage IV	17 (30.9)	42 (21.5)	60 (10.7)	0 (0.0)	0 (0.0)	0 (0.0)
Outcomes						
Median follow‐up, mon (min‐max)	50.2 (0.4‐111.3)	NA	NA	3.26 (0.14‐9.86)	38 (4‐112)	NA
No. of deaths, *n* (%)	20 (36.3)	84 (43.1)	NA	NA	NA	NA
OS, mon, mean ± SD	45.2 ± 23.4	48.6 ± 32.58	57.1 ± 38.5	NA	NA	NA
OS Events, *n* (%)	20 (36.3)	84 (43.1)	190 (33.8)	NA	NA	NA
DFS, mon, mean ± SD	34.7 ± 21.7	36.8 ± 32.7	48.9 ± 40.4	40.4 ± 26.5	47.7 ± 30.2	42.0 ± 23.4
DFS events, *n* (%)	15 (27.3)	53 (27.2)	177 (31.5)	18 (20.0)	8 (21.6)	37 (28.5)
Adjuvant Chemo, *n* (%)		NA		NA		NA
Yes	28 (50.9)		233 (41.5)		11 (29.7)	
No	9 (16.4)		329 (58.5)		26 (70.3)	
Unknown	18 (32.7)		0 (0.0)		0 (0.0)	
Tumor characteristics						
Mutations, *n* (%)	NA	NA		NA	NA	NA
KRAS			213 (37.9)			
TP53			190 (33.8)			
BRAF			51 (9.1)			
Side, *n* (%)						
Left	31 (56.4)	109 (55.9)	338 (60.1)	38 (42.2)	14 (37.8)	72 (55.4)
Right	16 (29.1)	86 (44.1)	220 (39.1)	52 (57.8)	23 (62.2)	57 (43.8)
Unknown	8 (14.5)	0 (0.0)	4 (0.7)	0 (0.0)	0 (0.0)	0 (0.0)
Grade, *n* (%)			NA			NA
1, well differentiated	1 (2.0)	17 (8.7)		61 (67.8)	8 (21.6)	
2, moderately differentiated	25 (45.5)	147 (75.4)			21 (56.8)	
3, poorly differentiated	3 (5.5)	31 (15.9)		29 (32.2)	6 (16.2)	
Unknown	26 (47.3)	0 (0.0)		0 (0.0)	2 (5.4)	

Abbreviations: MCC, Moffit Cancer Center; NA, Not Available; VUMC, Vanderbilt University Medical Center.

#### 2.5.2 Validation of the prediction model

The accuracy of the SMAD4‐modulated gene profile in predicting DFS was assessed as follows. The centroid of each cluster in the training data (GSE39582) was used to assign cluster membership to each tumor in the validation dataset (GSE33113, GSE31595, and GSE37892) using Prediction Analysis for Microarrays (PAM) in the R package *pamr* ^44^ (R version 3.5.2; Data S1). Batch effect in the validation cohort was removed using *ComBat* function from Bioconductor package *sva* before applying PAM prediction. Kaplan‐Meier analysis was then used to determine if the differences in DFS between the predicted high‐ and low‐risk clusters in the validation dataset were similar to those in the training dataset.

#### 2.5.3 Statistical methods

The Robust Multi‐Array Average algorithm in the Bioconductor *Affy* package was applied to preprocess and normalize Affymetrix microarray datasets. Association with available clinical variables was tested using Pearson's chi‐squared test. Spearman correlation was used to examine BMP/Wnt combined list with SMAD4 expression level, and the raw *p*‐values were adjusted for multiple comparisons using Benjamini‐Hochberg procedure.^45^ Prediction analysis for microarrays (PAM) in the R package *pamr* was applied to build a risk prediction model. Kaplan‐Meier analysis and log‐rank test were used to determine the differences in DFS between the predicted high‐ and low‐risk clusters. Associations with stage, sex, location, CpG island methylator phenotype status, chromosomal instability status, mismatch repair status, and mutational status of TP53, KRAS, or BRAF were tested, when available, using the Pearson chi‐squared test with the Yates continuity correction. Association with age was analyzed using the Wilcoxon rank‐sum test with continuity correction.

## 3 RESULTS

### 3.1 SMAD4‐modulated gene profile

**FIGURE 2:**
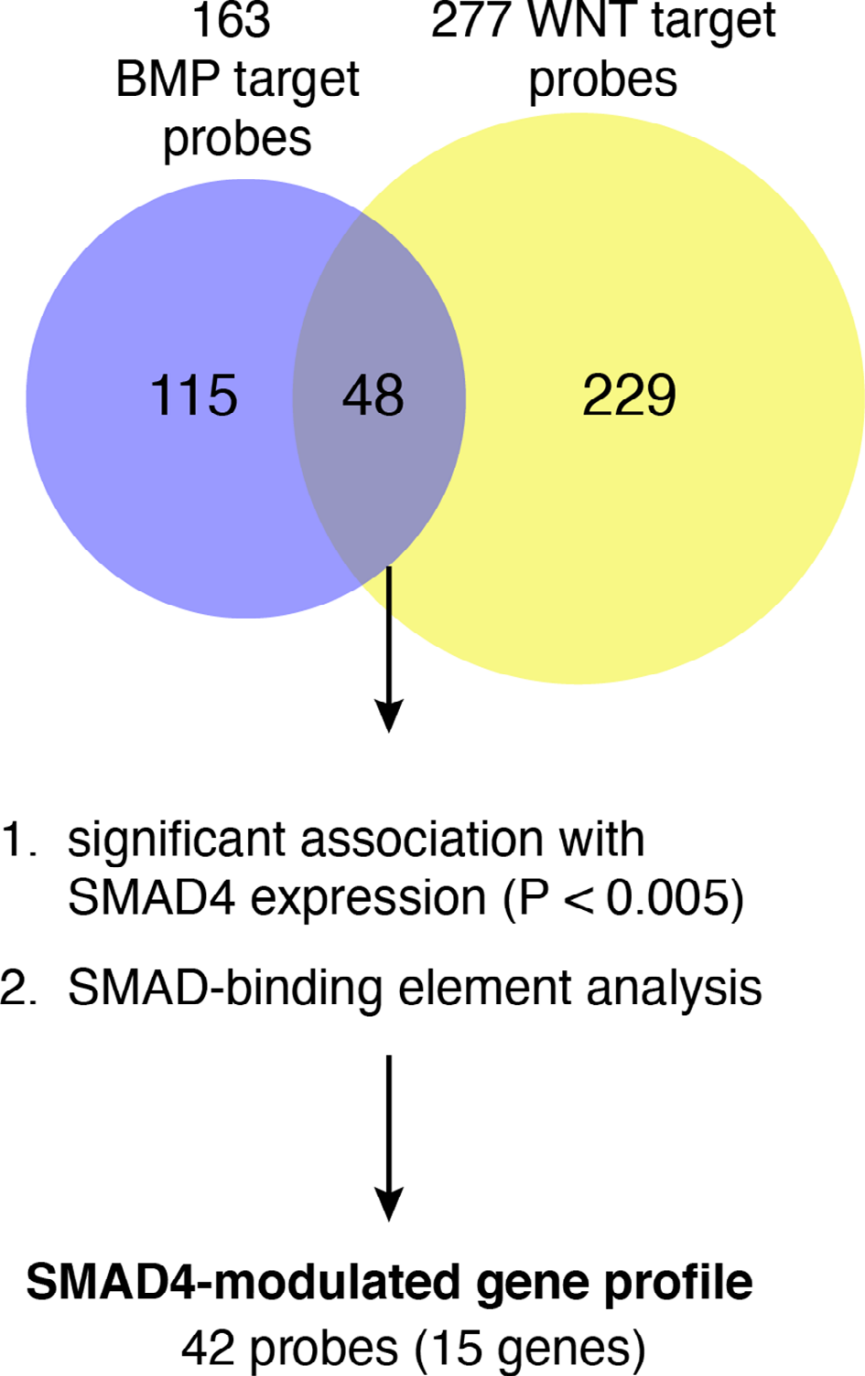
Experimental approach used to identify the SMAD4‐modulated gene profile. Lists of BMP and Wnt target genes were generated, and the overlap (48 probes) is shown in a Venn diagram. These overlapping genes/probes were tested for correlation with SMAD4 expression levels (Spearman correlation *p* < .005) and for SMAD‐binding elements in their promoters (see Methods). The SMAD4‐modulated gene profile was defined as genes/probes that passed either of these tests

The general flow of the study is represented in Figure 1. We compiled lists of BMP and Wnt target genes as described in the Methods. These genes corresponded to 163 BMP and 277 Wnt array probes (Figure 2) on the Affymetrix platform. After identifying 48 probes common to the two lists, we used a two‐stage approach to identify a SMAD4‐modulated gene profile (Figure 2). First, we identified BMP/Wnt genes whose expression was significantly associated (Spearman correlation FDR < 0.01) with SMAD4 expression in a discovery dataset of transcriptomic data of 250 CRC patients from VUMC and MCC (GSE161158).^25^ This analysis implicated 27 probes or 13 genes. Second, to interrogate putative SMAD4 activity in the BMP/Wnt gene list, we identified genes from the BMP/Wnt list containing SBEs in their promoter regions and then determined which corresponding probes were correlated with SMAD4 expression in the discovery dataset. Using this combined approach, we implicated 42 probes, or 15 distinct genes (Figure 2; Table S5). These 15 genes were defined as the SMAD4‐modulated gene profile. Fourteen of the 15 implicated genes contain known SBEs in their promoter regions.^33^ Five of the genes (e.g., *TCF7* and *MYC*) had expressions negatively correlating with SMAD4 (e.g., were putatively downregulated by SMAD4), while the other 10 (e.g., *DKK1* and *JAG1*) had expressions positively correlating with SMAD4 (see Table 1).

### 3.2 Biologic validation of target genes

**FIGURE 3:**
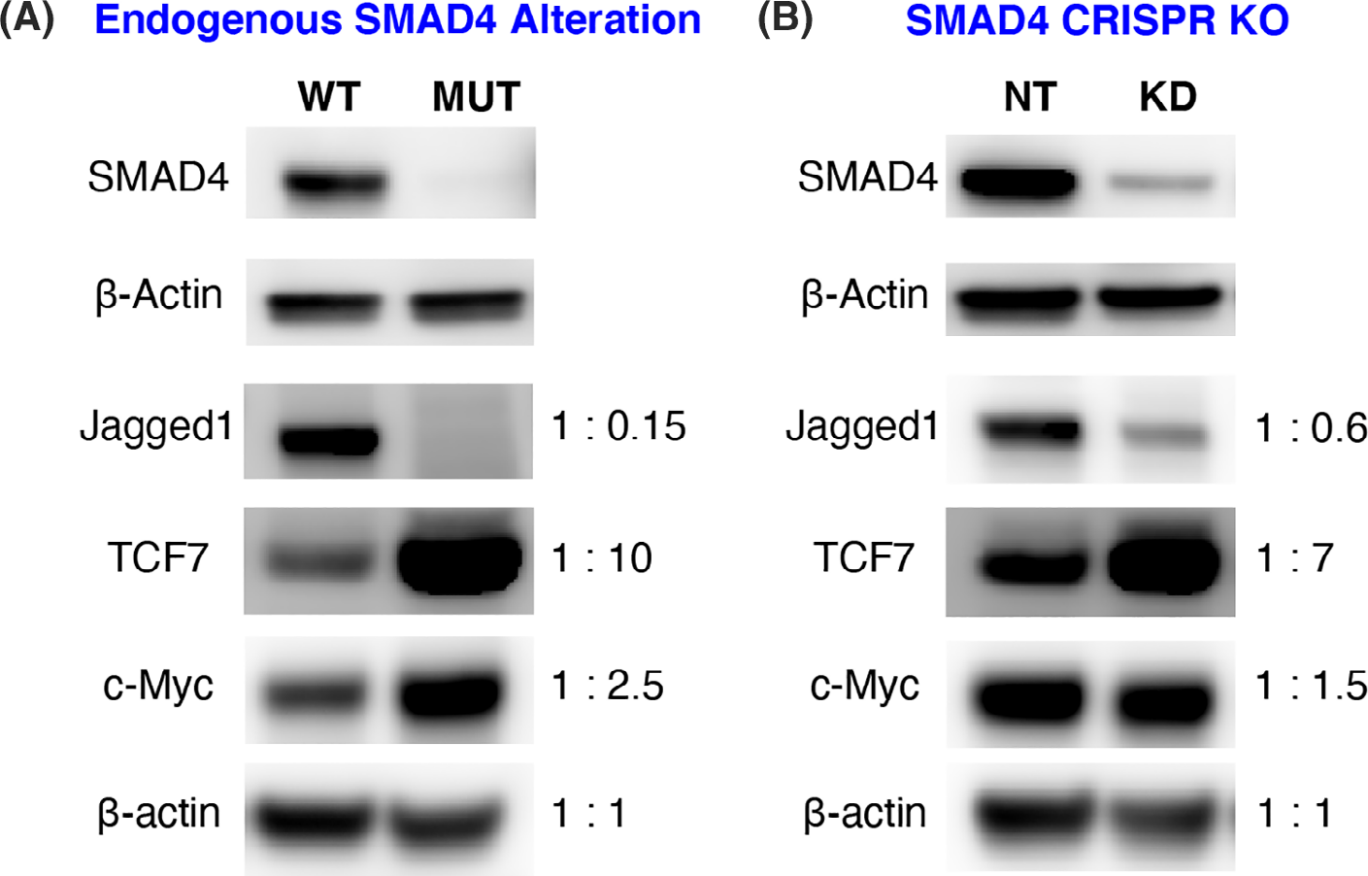
Validation of crosstalk between SMAD4 and target genes. (A) Tumoroid line derived from a SMAD4 wild‐type (WT) CRC patient and a tumoroid line derived from a SMAD4 mutant (MUT) CRC patient. Western blot shows SMAD4 status and inverse correlations between SMAD4 and three target genes: JAG1, TCF7, and c‐Myc. (B) Crispr/Cas9 technology was used to deplete SMAD4 in CRC patient‐derived tumor organoids (tumoroids). Compared to non‐targeted control (NT), western blot shows 80% SMAD4 knockdown (KD) and an inverse correlation between SMAD4, JAG1, TCF7, and c‐Myc with engineered depletion of SMAD4. For A and B, each quantification was normalized to the loading control β‐Actin. The respective fold‐change after normalization is shown for each condition in A and B to the right of the western blot

We next asked whether we could biologically validate select targets *in vitro* using three‐dimensional (3D) tumoroid models derived from CRC patients. We examined the three proteins from our SMAD4‐modulated profile for which reliable antibodies were available (Jagged‐1 [encoded by *JAG1*], TCF7, and c‐MYC) and assessed their levels in CRC tumoroids with and without SMAD4. Specifically, we compared a CRC tumoroid line derived from a *SMAD4* wild‐type tumor to a CRC tumoroid line derived from a *SMAD4* mutant tumor identified by MSK‐IMPACT sequencing.^46^ In parallel, and to ensure that any differences observed were not due to unknown mutations that may have varied between the lines, a separate *SMAD4* wild type, patient‐derived organoid (tumoroid) line was depleted of SMAD4 using CRISPR/Cas9‐mediated excision of the *SMAD4* gene. Western blot analysis showed that Jagged‐1 levels decreased with SMAD4 loss (Figure 3 (A, B)), consistent with the predicted positive regulation of *JAG1* by SMAD4 (see Table 1). In addition, levels of TCF7 and MYC were upregulated in the CRC tumoroids with *SMAD4* mutation compared to wild type and thus inversely correlated with SMAD4 levels (Figure 3 (A, B)). These findings thus provide biological evidence of modulation of the target genes *TCF7*, *MYC*, and *JAG1* in non‐engineered and engineered CRC tumoroid lines and demonstrate directional consistency based on SMAD4 status as predicted in Table 1.

### 3.3 The SMAD4‐modulated gene profile identifies patients with high‐risk stage II or III CRC

**FIGURE 4:**
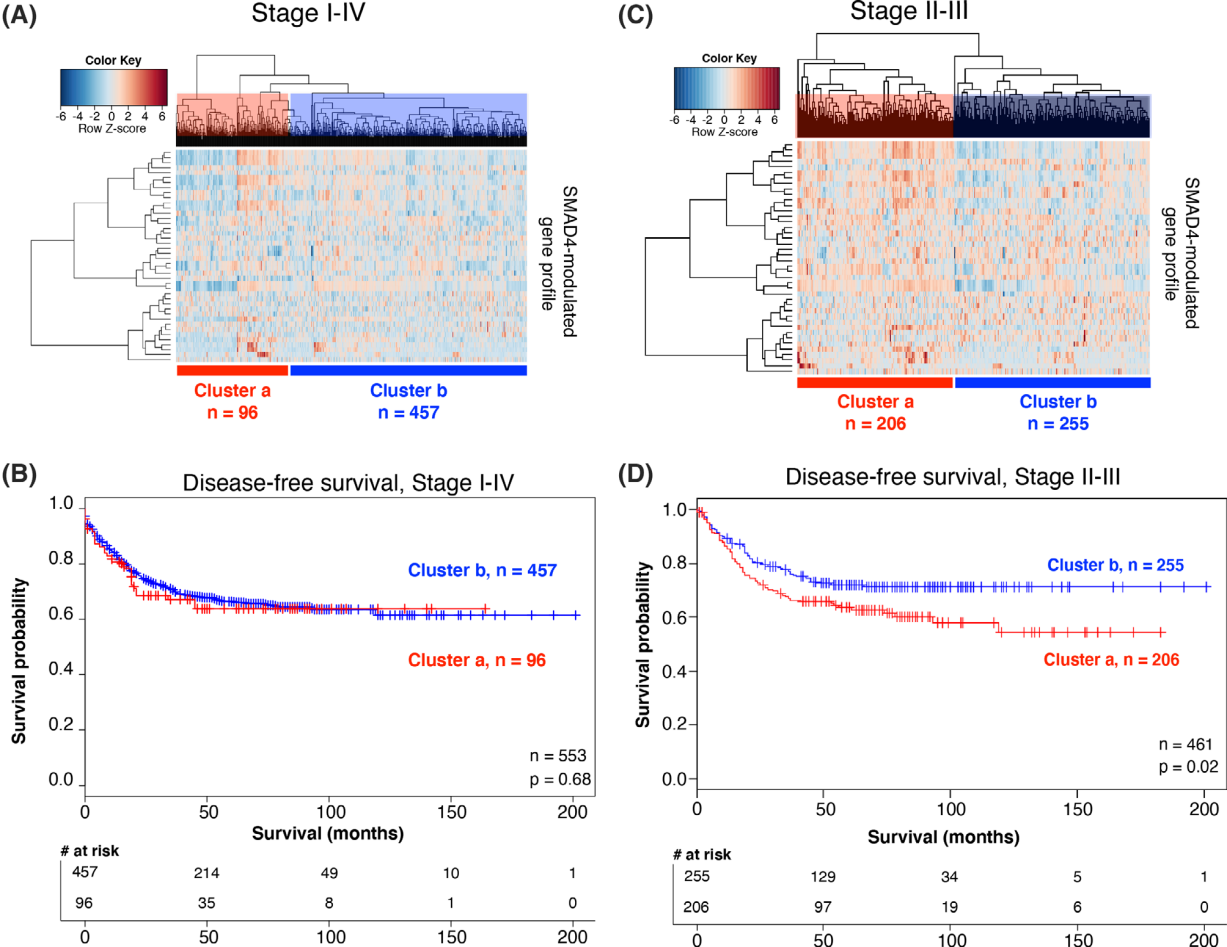
The SMAD4‐modulated gene profile is not associated with DFS in stage I‐IV CRC patients. (A) In the training dataset of 553 stage I‐IV CRC patients, two distinct patient clusters (Cluster a, red; Cluster b, blue) were identified via unsupervised hierarchical clustering. Each row on the heat map represents a single probe in the mean‐centered gene profile, and each column represents an individual patient in the training dataset. (B) Kaplan‐Meier analysis showed no significant difference in DFS between the clusters (DFS probability at 5 years: cluster a 63.8% and cluster b 66.5%; *p* = .68). (C) In the subset of 461 stage II and III CRC patients in the training dataset, two distinct patient clusters (Cluster a, red; Cluster b, blue) were identified in unsupervised hierarchical clustering. Each row on the heat map represents a single probe in the mean‐centered gene profile, and each column represents an individual patient. (D) Kaplan‐Meier analysis showed a significantly higher DFS in Cluster b patients than in Cluster a patients (75% vs. 58% at 100 months; *p* = .02)

After generating the SMAD4‐modulated gene profile using data from the discovery cohort, we assessed whether this gene profile could be used to identify CRC patients at risk of recurrent disease and corresponding worse outcomes. We used the SMAD4‐modulated gene profile to examine stage I‐IV CRC patients in a separate training dataset of 553 patients (GSE39582),^24^ which is the largest CRC patient microarray dataset available in the GEO repository. Unsupervised hierarchical clustering identified two distinct patient clusters that differed in the expression levels of the SMAD4‐modulated genes (Cluster a, *n* = 96; Cluster b, *n* = 457; Figure 4(A)). However, Kaplan‐Meier analysis showed no statistically significant differences in DFS between the two clusters (log‐rank test, *p* = .68, Figure 4(B)).

Because current diagnostic and prognostic measures have been unsuccessful in accurately identifying high‐risk stage II and III patients,^5^, ^6^, ^47^ we next used the SMAD4‐modulated gene profile to examine only the 461 stage II and III patients in the training dataset. Unsupervised hierarchical clustering revealed two distinct patient clusters (Figure 4(C)). In contrast to the finding from analyzing the full cohort, the stage II and III subset analysis revealed a significantly lower DFS in Cluster a (*n* = 206) than in Cluster b (*n* = 255) (Figure 4(D), median survival time not yet reached, *p* = .02). We found no association of cluster with gender, age, stage, CpG island methylator phenotype status, chromosomal instability status, mismatch repair status, or mutational status of *TP53*, *KRAS*, or *BRAF*. However, we found that there were more node‐positive patients in Cluster a than in Cluster b (*p* = .003) and more hindgut tumors in Cluster b than in Cluster a (66% vs. 52%, respectively; *p* = .004). Interestingly, *SMAD4* mRNA levels alone (median or quartile expression cutoffs) were not associated with DFS in either the full cohort or the stage II and III subset of patients.

### 3.4 The SMAD4‐modulated gene profile predicts DFS

**FIGURE 5:**
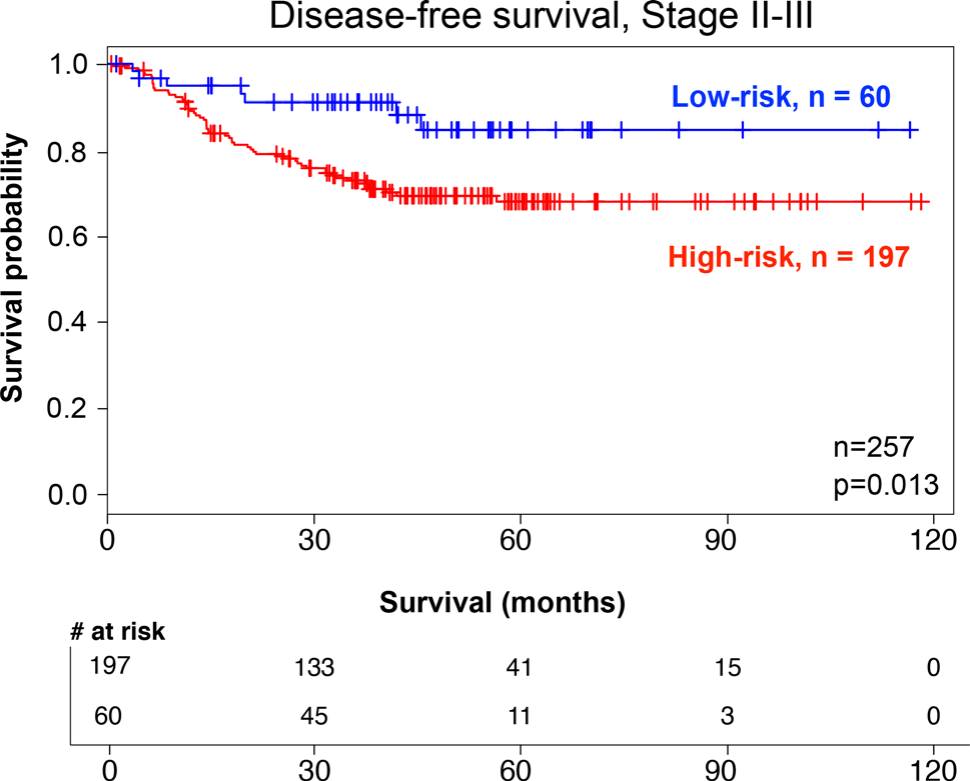
SMAD4‐modulated gene profile predicts DFS in stage II/III CRC patients in the validation dataset. A prediction model based on the SMAD4‐modulated gene profile identified 60 low‐ and 197 high‐risk patients in the validation dataset of stage II and III CRC patients (probability of DFS at 5 years: 67.7% in the high‐risk cluster vs. 84.6% in the low‐risk cluster; *p* = .013)

To confirm the association between the SMAD4‐modulated gene profile and DFS in stage II and III patients, we generated a centroid prediction model based on the SMAD4 profile and then investigated its predictive accuracy in a validation dataset of stage II and III CRC patients (*n* = 257; Table S6). The patients identified on the basis of the gene profile as low risk (*n* = 60) had a significantly higher 5‐year DFS rate than the patients identified as high risk (*n* = 197) (84.6% vs. 67.7%, respectively; *p* = .013) (Figure 5). The low‐risk and high‐risk groups did not differ significantly in age, sex, or tumor location (hindgut/midgut). The two groups did not differ in the proportion of stage II and III patients represented. Information regarding node status was unavailable for these datasets.

### 3.5 Neither the BMP nor the Wnt gene profile alone identifies high‐risk stage II/III CRC patients

We also investigated if either the BMP or the Wnt gene profile alone could identify patients with high‐risk stage II or III CRC. Using the BMP and Wnt target lists (Tables S1 and S2), we followed a similar methodologic progression as above. Specifically, we generated a centroid prediction model based on the stage II and III patients in our discovery cohort (*n* = 461) and then investigated its predictive accuracy in a validation dataset (*n* = 257). In the validation dataset, Kaplan‐Meier analysis showed that neither profile was associated with DFS (Figure S1).

## 4 DISCUSSION

The identification of patients who would benefit from adjuvant chemotherapy after surgical resection or who would likely to be cured by surgical resection alone has been elusive. With the aim of better stratifying patients by risk, we investigated whether patients at high risk of recurrence could be identified by a SMAD4‐modulated profile. This profile is based on previous biological findings that demonstrate upregulation of canonical Wnt signaling induced by loss of SMAD4 or inhibition of BMP,^16^ and the profile is supported by biological validation of several gene products in a tumoroid CRC model. While this profile was not successful in stratifying patients by DFS when examining stage I‐IV patients, subgroup analysis of the SMAD4‐modulated gene expression‐based profile in stage II and III patients successfully identified high‐risk individuals with worse DFS in both training and validation cohorts. Interestingly, neither SMAD4 expression level alone, a BMP gene profile, nor a Wnt gene profile alone could predict DFS.

A number of studies have shown that loss of SMAD4 protein is associated with worse outcomes in CRC patients, but to our knowledge, this is the first RNA‐based, quantitative predictor related to SMAD4. This SMAD4‐modulated gene profile will need to be examined in a prospective cohort of CRC patients before it can be adapted to a clinical setting. Nevertheless, our investigation is an initial step in the development of a surrogate tool for identifying high‐risk CRC patients based on resected specimens. Furthermore, given that SMAD4 loss assessed by IHC is associated with resistance to 5‐fluorouracil‐based therapy,^12^, ^13^, ^48^ future research will examine how our SMAD4‐modulated gene profile may predict chemoresistance. With the current lack of tools to identify high‐risk stage II and III CRC patients and the potential morbidity and costs associated with chemotherapy, a gene expression‐based profile that identifies high‐risk stage II and III patients will likely prove useful in selecting better treatment options based on prognostic genetic variables. Therefore, in our future work, we plan to prospectively collect specimens to determine the utility of the SMAD4‐modulated gene expression‐based signature in predicting response to various chemotherapeutic regimens currently in use. This signature may also serve as a proxy for IHC or mutational status^49^ in measuring SMAD4 loss. Currently, we are constructing a gene‐based tool that could be used prospectively on formalin‐fixed paraffin‐embedded tissue samples from another independent patient cohort with stage II and III CRC.

Our study has also uncovered a gene program modulated by SMAD4 in sporadic CRCs in which the primary defect is upregulation of the deeply conserved Wnt pathway. We demonstrated that SMAD4 modulates expression of key BMP/Wnt target genes, including *MYC*, *TCF7*, and *JAG1*. Our MYC findings are consistent with previous work from others^50^ and our group^16^ in which SMAD4 depletion in an epithelial‐specific manner induced significant upregulation of Myc RNA in murine models. Furthermore, one study investigating SMAD4 loss in a mouse model of inflammation‐associated CRC correlated five genes in our profile with SMAD4 knockout (*MYC*, *TCF4*, *DKK1*, *BTRC*, and *ID2*).^51^ Additional studies have investigated the SMAD4 transcriptional program in normal cells^52^ and cervical cancer cells,^53^ but none has investigated it in the context of a specific subset of CRC patients. We acknowledge that gene expression profiles associated with TGF‐β and Wnt have implicated a putative TGF‐β inhibitor, BAMBI, in patients with metastatic CRC^29^ and identified the involvement of another Wnt target, TCF4, in transformation of human epithelial cells,^30^ but these profiles used a different approach and did not implicate SMAD4. We have also developed and validated patient‐derived CRC tumoroid models for further investigation of the processes underlying tumor progression and resistance to therapy in association with SMAD4 loss. These tumoroid models will facilitate investigation of the complex transcriptional mechanisms involved in the loss of tumor suppression in association with SMAD4 loss in ongoing studies. Using this model, we plan to test compounds that could potentially recapitulate the signature (e.g., coordinately inhibit or activate proteins encoded by signature genes) in vivo and in vitro in order to elucidate the underlying biologic mechanism.

Our study has some limitations. Detailed pathology reports were not available for the multiple datasets used, which would have allowed a more complete examination of associations of the SMAD4 target profile with known high‐risk features. It is also possible that our BMP or Wnt target gene lists omitted some target genes because they were unknown at the time of our study. Although other important genes that are not BMP or Wnt targets could be regulated by SMAD4, our focus on BMP and Wnt targets was based on a strong biological rationale^16^ In addition, our use of the Affymetrix platform may limit generalizability to other platforms. It was also not possible to confirm via IHC or mutational status whether SMAD4 mRNA levels were correlated with SMAD4 protein levels or with SMAD4 mutation in the training and validation datasets. We are addressing this possibility in our ongoing work, as it may provide important insights into the biology of SMAD4 in CRC. Lastly, without complete treatment data for either the training cohort or the validation cohort, we were unable to examine associations between the profile and chemoresistance in this study. However, this study is greatly strengthened by its rational biologic design, the potentially wide applicability of the SMAD4‐modulated profile given its validation in an independent cohort, and the biologic validation of molecular targets in *in vitro* models.

## 5 CONCLUSION

Using a biologically informed perspective to derive a SMAD4‐modulated gene profile, we validated a prognostic model to identify low‐ and high‐risk groups of stage II/III CRC patients on the basis of expression of SMAD4‐modulated genes. This gene profile has potential for prognostic use in select CRC patients.

## CONFLICT OF INTEREST

J. Joshua Smith has received travel support for fellow education (2015) from Intuitive Surgical Inc. J. Joshua Smith also served as clinical advisor (2019) for Guardant Health, Inc. Julio Garcia‐Aguilar has received support from Medtronic (honorarium for consultancy with Medtronic), Johnson & Johnson (honorarium for delivering a talk), and Intuitive Surgical (honorarium for participating in a webinar by Intuitive Surgical Inc.). All other authors declare that they have no competing interests.

## ETHICAL STATEMENT

Use of patient‐derived tissues and subsequentexperiments at MSK were approved by the Institutional Review Board under an approved protocol (Dr. Smith ‐ PI). Patients also consented for tissue use and sequencing on a separate protocol.

## AUTHORS' CONTRIBUTIONS

All authors had full access to the data in the study and take responsibility for the integrity of the data and the accuracy of the data analysis. *Conceptualization*, R.D.B, X.S.C, J.J.S.; *Data Curation*, B.C.S., C.W., M.R.M., H.S.P., Z.Z., B.Z., X.S.C., J.J.S.; *Methodology*, R.D.B., X.S.C., J.J.S.; *Investigation*, B.C.S., C.W., J.J.S.; *Formal Analysis*, M.R.M., H.S.P., Z.Z., X.S.C., J.J.S.; *Resources*, J.G.‐A., J.J.S.; *Writing ‐ Original Draft*, B.C.S., C.W., R.D.B., X.S.C., J.J.S.; *Writing ‐ Review & Editing*, B.C.S., C.W., J.G.‐A., R.D.B., X.S.C., J.J.S.; *Project Administration*, J.G.‐A., R.D.B., J.J.S.; *Visualization*, B.C.S., X.S.C., J.J.S.; *Supervision*, J.G.‐A.,J.J.S.; *Funding Acquisition*, J.G.‐A., R.D.B., X.S.C., J.J.S.; *Software*, X.S.C.; *Validation*, X.S.C., J.J.S.

## Supporting information


**Figure S1.** Neither the BMP nor the Wnt profile alone is associated with DFS in stage II and III patients in the validation dataset. (A, C) Two patient clusters (cluster a, red; cluster b, blue) were observed in unsupervised hierarchical clustering using either BMP or Wnt signature. Rows represent mean‐centered gene profiles of BMP and Wnt signatures, respectively, and columns represent individual patients in the validation dataset. However, (B, D) Kaplan‐Meier analysis revealed that there is no significant difference in DFS between the clusters using either BMP or Wnt expression profile (*n* = 257).

**Table S1.** BMP target gene list. Gene ontology tool was used to generate the list. The list was validated by manual search and verification in PubMed. *n* = 66 genes (163 probes)

**Table S2.** Wnt target gene list. Gene ontology tool was used to generate the list. The list was validated by manual search and verification in PubMed. *n* = 112 genes (277 probes)

**Table S3.** BMP target probe identifiers.

**Table S4.**. Wnt target probe identifiers.

**Table S5.** SMAD4 probe list.

**Table S6.** Centroid values.

**Data S1.** Supplement R file.

## Data Availability

The discovery dataset (VUMC and MCC) is deposited in the Gene Expression Omnibus (GEO) repository at GSE161158. The other datasets used in this study are also available publicly through the GEO repository as follows: Training: Dataset, GSE39582^24^; Validation: GSE33113,^39^ GSE31595,^40^ GSE37892.^41^
