# Supplementary material for: A SMAD4‐modulated gene profile predicts disease‐free survival in stage II and III colorectal cancer
Source: Cancer Rep (Hoboken). 2021 Jun 10;5(1):e1423. doi: 10.1002/cnr2.1423 (PMC8789617; doi:10.1002/cnr2.1423)
Supplement: Supplementary file 1 — Figure S1. Neither the BMP nor the Wnt profile alone is associated with DFS in stage II and III patients in the validation dataset. (A, C) Two patient clusters (cluster a, red; cluster b, blue) were observed in unsupervised hierarchical clustering using either BMP or Wnt signature. Rows represent mean‐centered gene profiles of BMP and Wnt signatures, respectively, and columns represent individual patients in the validation dataset. However, (B, D) Kaplan‐Meier analysis revealed that there is no significant difference in DFS between the clusters using either BMP or Wnt expression profile (n = 257). [file CNR2-5-e1423-s006.pdf]

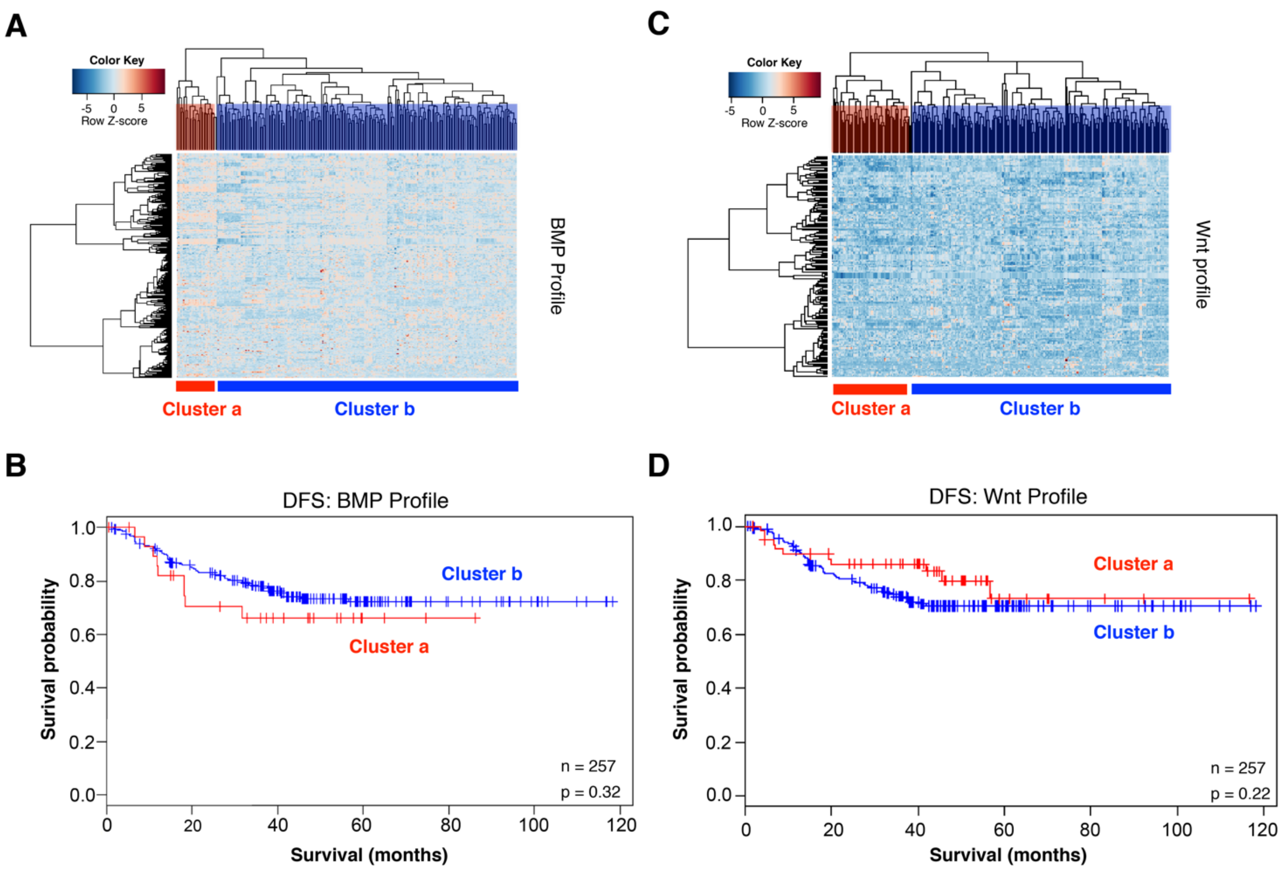

Figure S1. Neither the BMP nor the Wnt profile alone is associated with DFS in stage II and III patients in the validation dataset. (A, C) Two patient clusters (cluster a, red; cluster b, blue) were observed in unsupervised hierarchical clustering using either BMP or Wnt signature. Rows represent mean-centered gene profiles of BMP and Wnt signatures, respectively, and columns represent individual patients in the validation dataset. However, (B, D) Kaplan-Meier analysis revealed that there is no significant difference in DFS between the clusters using either BMP or Wnt expression profile (n = 257).
