# Supplementary material for: A SMAD4‐modulated gene profile predicts disease‐free survival in stage II and III colorectal cancer
Source: Cancer Rep (Hoboken). 2021 Jun 10;5(1):e1423. doi: 10.1002/cnr2.1423 (PMC8789617; doi:10.1002/cnr2.1423)
Supplement: Supplementary file 2 — Table S1. BMP target gene list. Gene ontology tool was used to generate the list. The list was validated by manual search and verification in PubMed. n = 66 genes (163 probes) [file CNR2-5-e1423-s005.pdf]

Table S1: BMP target gene list. Gene ontology tool was used to generate the list.
the list was validated by manual search and verification in PubMed. n = 66 genes (163 probes)

ADCYAP1	GATA3	MSX1	SOCS3
ATOH8	HAND1	MSX2	SOST
BAMBI	HAND2	MSX2	SOX4
BCOR	HES1	MYC	SOX9
BMPR2	HEY1	NFKBIA	TBX6
BTRC	ID1	NGFR	TCF4
CDX2	ID2	NIPSNAP1	TCF7
CXCR4	ID3	NOG	TJP1
CXXC5	ID4	PPARG	TWIST1
DKK1	IHH	PSIP1	VEGFA
DLL1	IRF8	S100P	VIP
DLX2	JAG1	S1PR1	WIF1
ENG	JUNB	S1PR2	WNT1
FGFR2	KLF10	SMAD6	WNT4
FN1	KRT15	SMAD7	WNT8A
FST	LEF1	SNAI1	
GATA2	LRP5	SNAI2	
