# Supplementary material for: A SMAD4‐modulated gene profile predicts disease‐free survival in stage II and III colorectal cancer
Source: Cancer Rep (Hoboken). 2021 Jun 10;5(1):e1423. doi: 10.1002/cnr2.1423 (PMC8789617; doi:10.1002/cnr2.1423)
Supplement: Supplementary file 3 — Table S2. Wnt target gene list. Gene ontology tool was used to generate the list. The list was validated by manual search and verification in PubMed. n = 112 genes (277 probes) [file CNR2-5-e1423-s001.pdf]

Table S2: Wnt target gene list. Gene ontology tool was used to generate the list. the list was validated by manual search and verification in PubMed. n = 112 genes (277 probes)

ABCB1	EDA	HOXB9	MYC	SOX2
ATOH1	EDN1	ID2	MYCN	SOX9
AXIN2	EFNB1	IGF1	MYOD1	SP5
BIRC5	EFNB2	IL6	NANOG	STRA6
BMP10	EFNB3	IRX3	NEUROG1	TCF4
BMP15	EGFR	ISL1	NKX2-2	TCF7
BMP2	EN1	ISLR	NOS2	TIAM1
BMP4	EN2	JAG1	NRCAM	TNFRSF11A
BMP7	ENPP2	JUN	PITX2	TNFRSF19
BMP8A	FGF18	KLF4	PLAUR	TNFRSF9
BMP8B	FGF20	KLF5	POSTN	TWIST1
BTRC	FGF4	KRT5	PPARD	UBXN1
CCND1	FGF9	L1CAM	PTGS2	UBXN10
CD44	FN1	LEF1	PTTG1	UBXN8
CDH1	FOSL1	LGR5	RARG	VCAN
CDKN2A	FOXN1	MAEA	RET	VEGFA
CDX1	FST	MET	RHOU	VEGFC
CDX4	FZD7	MITF	RUNX2	WISP1
CLDN1	GAST	MMP2	SALL4	WISP2
CTLA4	GCG	MMP26	SFRP2	WNT1
CYR61	GJA1	MMP3	SIX3	
DKK1	GJB6	MMP7	SNAI1	
DLL1	GREM2	MMP9	SNX9	
