# Supplementary material for: A SMAD4‐modulated gene profile predicts disease‐free survival in stage II and III colorectal cancer
Source: Cancer Rep (Hoboken). 2021 Jun 10;5(1):e1423. doi: 10.1002/cnr2.1423 (PMC8789617; doi:10.1002/cnr2.1423)
Supplement: Supplementary file 4 — Table S3. BMP target probe identifiers. [file CNR2-5-e1423-s004.pdf]

Table S3: BMP target probe identifiers.

	probelist	geneSymbol
1554933_at	1554933_at	PSIP1
1556075_at	1556075_at	BMPR2
1556689_a_at	1556689_a_at	WNT4
1558199_at	1558199_at	FN1
1558705_at	1558705_at	ATOH8
1558706_a_at	1558706_a_at	ATOH8
1563620_at	1563620_at	BTRC
1567906_at	1567906_at	SOX4
201416_at	201416_at	SOX4
201417_at	201417_at	SOX4
201418_s_at	201418_s_at	SOX4
201473_at	201473_at	JUNB
201502_s_at	201502_s_at	NFKBIA
201565_s_at	201565_s_at	ID2
201566_x_at	201566_x_at	ID2
201708_s_at	201708_s_at	NIPSNAP1
201709_s_at	201709_s_at	NIPSNAP1
201808_s_at	201808_s_at	ENG
201809_s_at	201809_s_at	ENG
202011_at	202011_at	TJP1
202393_s_at	202393_s_at	KLF10
202431_s_at	202431_s_at	MYC
202935_s_at	202935_s_at	SOX9
202936_s_at	202936_s_at	SOX9
203304_at	203304_at	BAMBI
203393_at	203393_at	HES1
203394_s_at	203394_s_at	HES1
203395_s_at	203395_s_at	HES1
203638_s_at	203638_s_at	FGFR2
203639_s_at	203639_s_at	FGFR2
203753_at	203753_at	TCF4
204057_at	204057_at	IRF8
204351_at	204351_at	S100P
204602_at	204602_at	DKK1
204642_at	204642_at	S1PR1
204712_at	204712_at	WIF1
204734_at	204734_at	KRT15
204790_at	204790_at	SMAD7
204901_at	204901_at	BTRC
204948_s_at	204948_s_at	FST
205254_x_at	205254_x_at	TCF7
205255_x_at	205255_x_at	TCF7
205555_s_at	205555_s_at	MSX2
205556_at	205556_at	MSX2
205858_at	205858_at	NGFR
205932_s_at	205932_s_at	MSX1

205961_s_at	205961_s_at	PSIP1
206281_at	206281_at	ADCYAP1
206359_at	206359_at	SOCS3
206360_s_at	206360_s_at	SOCS3
206387_at	206387_at	CDX2
206577_at	206577_at	VIP
207069_s_at	207069_s_at	SMAD6
207147_at	207147_at	DLX2
207345_at	207345_at	FST
207684_at	207684_at	TBX6
207826_s_at	207826_s_at	ID3
207954_at	207954_at	GATA2
208225_at	208225_at	FGFR2
208228_s_at	208228_s_at	FGFR2
208229_at	208229_at	FGFR2
208234_x_at	208234_x_at	FGFR2
208510_s_at	208510_s_at	PPARG
208537_at	208537_at	S1PR2
208570_at	208570_at	WNT1
208606_s_at	208606_s_at	WNT4
208937_s_at	208937_s_at	ID1
209097_s_at	209097_s_at	JAG1
209098_s_at	209098_s_at	JAG1
209099_x_at	209099_x_at	JAG1
209201_x_at	209201_x_at	CXCR4
209291_at	209291_at	ID4
209292_at	209292_at	ID4
209293_x_at	209293_x_at	ID4
209337_at	209337_at	PSIP1
209468_at	209468_at	LRP5
209602_s_at	209602_s_at	GATA3
209603_at	209603_at	GATA3
209604_s_at	209604_s_at	GATA3
209710_at	209710_at	GATA2
209886_s_at	209886_s_at	SMAD6
209887_at	209887_at	SMAD6
209920_at	209920_at	BMPR2
210214_s_at	210214_s_at	BMPR2
210319_x_at	210319_x_at	MSX2
210358_x_at	210358_x_at	GATA2
210495_x_at	210495_x_at	FN1
210512_s_at	210512_s_at	VEGFA
210513_s_at	210513_s_at	VEGFA
210758_at	210758_at	PSIP1
210948_s_at	210948_s_at	LEF1
211398_at	211398_at	FGFR2
211399_at	211399_at	FGFR2

211400_at	211400_at	FGFR2
211401_s_at	211401_s_at	FGFR2
211527_x_at	211527_x_at	VEGFA
211719_x_at	211719_x_at	FN1
211919_s_at	211919_s_at	CXCR4
212171_x_at	212171_x_at	VEGFA
212382_at	212382_at	TCF4
212385_at	212385_at	TCF4
212386_at	212386_at	TCF4
212387_at	212387_at	TCF4
212464_s_at	212464_s_at	FN1
213139_at	213139_at	SNAI2
213565_s_at	213565_s_at	SMAD6
213665_at	213665_at	SOX4
213668_s_at	213668_s_at	SOX4
213891_s_at	213891_s_at	TCF4
213943_at	213943_at	TWIST1
214105_at	214105_at	SOCS3
214168_s_at	214168_s_at	TJP1
214701_s_at	214701_s_at	FN1
214702_at	214702_at	FN1
215122_at	215122_at	TBX6
215420_at	215420_at	IHH
215685_s_at	215685_s_at	DLX2
216091_s_at	216091_s_at	BTRC
216268_s_at	216268_s_at	JAG1
216442_x_at	216442_x_at	FN1
217028_at	217028_at	CXCR4
218839_at	218839_at	HEY1
219433_at	219433_at	BCOR
219480_at	219480_at	SNAI1
220138_at	220138_at	HAND1
220480_at	220480_at	HAND2
221557_s_at	221557_s_at	LEF1
221558_s_at	221558_s_at	LEF1
222146_s_at	222146_s_at	TCF4
222374_at	222374_at	BTRC
222996_s_at	222996_s_at	CXXC5
223566_s_at	223566_s_at	BCOR
223869_at	223869_at	SOST
223915_at	223915_at	BCOR
223916_s_at	223916_s_at	BCOR
224215_s_at	224215_s_at	DLL1
224259_at	224259_at	WNT8A
224471_s_at	224471_s_at	BTRC
224516_s_at	224516_s_at	CXXC5
225144_at	225144_at	BMPR2

226847_at	226847_at	FST
226933_s_at	226933_s_at	ID4
227684_at	227684_at	S1PR2
227697_at	227697_at	SOCS3
227938_s_at	227938_s_at	DLL1
228473_at	228473_at	MSX1
228586_at	228586_at	ENG
228837_at	228837_at	TCF4
228890_at	228890_at	ATOH8
229358_at	229358_at	IHH
229386_at	229386_at	ID4
229591_at	229591_at	LRP5
229924_s_at	229924_s_at	JAG1
230237_at	230237_at	ADCYAP1
230751_at	230751_at	WNT4
230842_at	230842_at	FGFR2
231699_at	231699_at	NFKBIA
231798_at	231798_at	NOG
231873_at	231873_at	BMPR2
233955_x_at	233955_x_at	CXXC5
238516_at	238516_at	BMPR2
240913_at	240913_at	FGFR2
44783_s_at	44783_s_at	HEY1
