# Supplementary material for: A SMAD4‐modulated gene profile predicts disease‐free survival in stage II and III colorectal cancer
Source: Cancer Rep (Hoboken). 2021 Jun 10;5(1):e1423. doi: 10.1002/cnr2.1423 (PMC8789617; doi:10.1002/cnr2.1423)
Supplement: Supplementary file 5 — Table S4.. Wnt target probe identifiers. [file CNR2-5-e1423-s003.pdf]

Table S4: Wnt target probe identifiers.

	probelist	geneSymbol
1552982_a_at	1552982_a_at	FGF4
1554874_at	1554874_at	MITF
1554997_a_at	1554997_a_at	PTGS2
1555777_at	1555777_at	POSTN
1555778_a_at	1555778_a_at	POSTN
1555826_at	1555826_at	BIRC5
1557905_s_at	1557905_s_at	CD44
1558199_at	1558199_at	FN1
1558687_a_at	1558687_a_at	FOXN1
1563620_at	1563620_at	BTRC
1564630_at	1564630_at	EDN1
1565483_at	1565483_at	EGFR
1565484_x_at	1565484_x_at	EGFR
1565868_at	1565868_at	CD44
1566677_at	1566677_at	MMP2
1566678_at	1566678_at	MMP2
1569334_at	1569334_at	STRA6
1569335_a_at	1569335_a_at	STRA6
201069_at	201069_at	MMP2
201130_s_at	201130_s_at	CDH1
201131_s_at	201131_s_at	CDH1
201289_at	201289_at	CYR61
201464_x_at	201464_x_at	JUN
201465_s_at	201465_s_at	JUN
201466_s_at	201466_s_at	JUN
201565_s_at	201565_s_at	ID2
201566_x_at	201566_x_at	ID2
201667_at	201667_at	GJA1
201820_at	201820_at	KRT5
201871_s_at	201871_s_at	UBXN1
201983_s_at	201983_s_at	EGFR
201984_s_at	201984_s_at	EGFR
202094_at	202094_at	BIRC5
202095_s_at	202095_s_at	BIRC5
202431_s_at	202431_s_at	MYC
202668_at	202668_at	EFNB2
202669_s_at	202669_s_at	EFNB2
202711_at	202711_at	EFNB1
202935_s_at	202935_s_at	SOX9
202936_s_at	202936_s_at	SOX9
203510_at	203510_at	MET
203554_x_at	203554_x_at	PTTG1
203705_s_at	203705_s_at	FZD7
203706_s_at	203706_s_at	FZD7
203753_at	203753_at	TCF4
203936_s_at	203936_s_at	MMP9

204105_s_at	204105_s_at	NRCAM
204188_s_at	204188_s_at	RARG
204189_at	204189_at	RARG
204259_at	204259_at	MMP7
204420_at	204420_at	FOSL1
204489_s_at	204489_s_at	CD44
204490_s_at	204490_s_at	CD44
204584_at	204584_at	L1CAM
204585_s_at	204585_s_at	L1CAM
204602_at	204602_at	DKK1
204619_s_at	204619_s_at	VCAN
204620_s_at	204620_s_at	VCAN
204748_at	204748_at	PTGS2
204901_at	204901_at	BTRC
204948_s_at	204948_s_at	FST
205031_at	205031_at	EFNB3
205207_at	205207_at	IL6
205254_x_at	205254_x_at	TCF7
205255_x_at	205255_x_at	TCF7
205289_at	205289_at	BMP2
205290_s_at	205290_s_at	BMP2
205792_at	205792_at	WISP2
205828_at	205828_at	MMP3
205879_x_at	205879_x_at	RET
206104_at	206104_at	ISL1
206217_at	206217_at	EDA
206404_at	206404_at	FGF9
206409_at	206409_at	TIAM1
206422_at	206422_at	GCG
206430_at	206430_at	CDX1
206634_at	206634_at	SIX3
206657_s_at	206657_s_at	MYOD1
206783_at	206783_at	FGF4
206796_at	206796_at	WISP1
206915_at	206915_at	NKX2-2
206986_at	206986_at	FGF18
206987_x_at	206987_x_at	FGF18
207037_at	207037_at	TNFRSF11A
207039_at	207039_at	CDKN2A
207060_at	207060_at	EN2
207191_s_at	207191_s_at	ISLR
207233_s_at	207233_s_at	MITF
207345_at	207345_at	FST
207536_s_at	207536_s_at	TNFRSF9
207558_s_at	207558_s_at	PITX2
207683_at	207683_at	FOXN1
207865_s_at	207865_s_at	BMP8B

207922_s_at	207922_s_at	MAEA
208044_s_at	208044_s_at	PPARD
208138_at	208138_at	GAST
208292_at	208292_at	BMP10
208497_x_at	208497_x_at	NEUROG1
208570_at	208570_at	WNT1
208711_s_at	208711_s_at	CCND1
208712_at	208712_at	CCND1
209097_s_at	209097_s_at	JAG1
209098_s_at	209098_s_at	JAG1
209099_x_at	209099_x_at	JAG1
209211_at	209211_at	KLF5
209212_s_at	209212_s_at	KLF5
209392_at	209392_at	ENPP2
209540_at	209540_at	IGF1
209541_at	209541_at	IGF1
209542_x_at	209542_x_at	IGF1
209590_at	209590_at	BMP7
209591_s_at	209591_s_at	BMP7
209644_x_at	209644_x_at	CDKN2A
209756_s_at	209756_s_at	MYCN
209757_s_at	209757_s_at	MYCN
209835_x_at	209835_x_at	CD44
209946_at	209946_at	VEGFC
209993_at	209993_at	ABCB1
210037_s_at	210037_s_at	NOS2
210334_x_at	210334_x_at	BIRC5
210393_at	210393_at	LGR5
210495_x_at	210495_x_at	FN1
210512_s_at	210512_s_at	VEGFA
210513_s_at	210513_s_at	VEGFA
210623_at	210623_at	UBXN1
210636_at	210636_at	PPARD
210764_s_at	210764_s_at	CYR61
210809_s_at	210809_s_at	POSTN
210839_s_at	210839_s_at	ENPP2
210845_s_at	210845_s_at	PLAUR
210883_x_at	210883_x_at	EFNB3
210916_s_at	210916_s_at	CD44
210948_s_at	210948_s_at	LEF1
210984_x_at	210984_x_at	EGFR
211029_x_at	211029_x_at	FGF18
211127_x_at	211127_x_at	EDA
211128_at	211128_at	EDA
211129_x_at	211129_x_at	EDA
211130_x_at	211130_x_at	EDA
211131_s_at	211131_s_at	EDA

211156_at	211156_at	CDKN2A
211259_s_at	211259_s_at	BMP7
211260_at	211260_at	BMP7
211312_s_at	211312_s_at	WISP1
211377_x_at	211377_x_at	MYCN
211421_s_at	211421_s_at	RET
211485_s_at	211485_s_at	FGF18
211518_s_at	211518_s_at	BMP4
211527_x_at	211527_x_at	VEGFA
211550_at	211550_at	EGFR
211551_at	211551_at	EGFR
211571_s_at	211571_s_at	VCAN
211577_s_at	211577_s_at	IGF1
211599_x_at	211599_x_at	MET
211607_x_at	211607_x_at	EGFR
211719_x_at	211719_x_at	FN1
211786_at	211786_at	TNFRSF9
211924_s_at	211924_s_at	PLAUR
212014_x_at	212014_x_at	CD44
212063_at	212063_at	CD44
212171_x_at	212171_x_at	VEGFA
212382_at	212382_at	TCF4
212385_at	212385_at	TCF4
212386_at	212386_at	TCF4
212387_at	212387_at	TCF4
212464_s_at	212464_s_at	FN1
213135_at	213135_at	TIAM1
213281_at	213281_at	JUN
213721_at	213721_at	SOX2
213722_at	213722_at	SOX2
213807_x_at	213807_x_at	MET
213816_s_at	213816_s_at	MET
213880_at	213880_at	LGR5
213891_s_at	213891_s_at	TCF4
213943_at	213943_at	TWIST1
214178_s_at	214178_s_at	SOX2
214284_s_at	214284_s_at	FGF18
214701_s_at	214701_s_at	FN1
214702_at	214702_at	FN1
214866_at	214866_at	PLAUR
214981_at	214981_at	POSTN
215646_s_at	215646_s_at	VCAN
215771_x_at	215771_x_at	RET
215983_s_at	215983_s_at	UBXN8
216056_at	216056_at	CD44
216091_s_at	216091_s_at	BTRC
216268_s_at	216268_s_at	JAG1

216417_x_at	216417_x_at	HOXB9
216442_x_at	216442_x_at	FN1
216959_x_at	216959_x_at	NRCAM
216994_s_at	216994_s_at	RUNX2
217178_at	217178_at	RARG
217523_at	217523_at	CD44
218182_s_at	218182_s_at	CLDN1
218995_s_at	218995_s_at	EDN1
219480_at	219480_at	SNAI1
220184_at	220184_at	NANOG
220203_at	220203_at	BMP8A
220204_s_at	220204_s_at	BMP8A
220266_s_at	220266_s_at	KLF4
220394_at	220394_at	FGF20
220541_at	220541_at	MMP26
220559_at	220559_at	EN1
220794_at	220794_at	GREM2
221282_x_at	221282_x_at	RUNX2
221283_at	221283_at	RUNX2
221331_x_at	221331_x_at	CTLA4
221332_at	221332_at	BMP15
221336_at	221336_at	ATOH1
221340_at	221340_at	CDX4
221557_s_at	221557_s_at	LEF1
221558_s_at	221558_s_at	LEF1
221701_s_at	221701_s_at	STRA6
221731_x_at	221731_x_at	VCAN
221841_s_at	221841_s_at	KLF4
222146_s_at	222146_s_at	TCF4
222374_at	222374_at	BTRC
222549_at	222549_at	CLDN1
222695_s_at	222695_s_at	AXIN2
222696_at	222696_at	AXIN2
222802_at	222802_at	EDN1
223027_at	223027_at	SNX9
223028_s_at	223028_s_at	SNX9
223121_s_at	223121_s_at	SFRP2
223122_s_at	223122_s_at	SFRP2
223168_at	223168_at	RHOU
223169_s_at	223169_s_at	RHOU
223827_at	223827_at	TNFRSF19
224090_s_at	224090_s_at	TNFRSF19
224176_s_at	224176_s_at	AXIN2
224215_s_at	224215_s_at	DLL1
224471_s_at	224471_s_at	BTRC
224498_x_at	224498_x_at	AXIN2
224999_at	224999_at	EGFR

226066_at	226066_at	MITF
226461_at	226461_at	HOXB9
226847_at	226847_at	FST
227812_at	227812_at	TNFRSF19
227938_s_at	227938_s_at	DLL1
228038_at	228038_at	SOX2
228837_at	228837_at	TCF4
229221_at	229221_at	CD44
229638_at	229638_at	IRX3
229661_at	229661_at	SALL4
229802_at	229802_at	WISP1
229924_s_at	229924_s_at	JAG1
230092_at	230092_at	UBXN10
231079_at	231079_at	NANOG
231382_at	231382_at	FGF18
231771_at	231771_at	GJB6
231794_at	231794_at	CTLA4
232109_at	232109_at	UBXN10
232231_at	232231_at	RUNX2
234362_s_at	234362_s_at	CTLA4
234376_at	234376_at	MYCN
234411_x_at	234411_x_at	CD44
234418_x_at	234418_x_at	CD44
234895_at	234895_at	CTLA4
235275_at	235275_at	BMP8B
235504_at	235504_at	GREM2
235821_at	235821_at	WISP1
235845_at	235845_at	SP5
236341_at	236341_at	CTLA4
236858_s_at	236858_s_at	RUNX2
236859_at	236859_at	RUNX2
238657_at	238657_at	UBXN10
238846_at	238846_at	TNFRSF11A
238950_at	238950_at	TNFRSF9
239178_at	239178_at	FGF9
240509_s_at	240509_s_at	GREM2
241072_s_at	241072_s_at	IGF1
242026_at	242026_at	MYCN
242054_s_at	242054_s_at	SIX3
242218_at	242218_at	PPARD
243951_at	243951_at	ABCB1
244288_s_at	244288_s_at	SIX3
37152_at	37152_at	PPARD
