# Supplementary material for: A SMAD4‐modulated gene profile predicts disease‐free survival in stage II and III colorectal cancer
Source: Cancer Rep (Hoboken). 2021 Jun 10;5(1):e1423. doi: 10.1002/cnr2.1423 (PMC8789617; doi:10.1002/cnr2.1423)
Supplement: Supplementary file 6 — Table S5. SMAD4 probe list. [file CNR2-5-e1423-s007.pdf]

Table S5: SMAD4 probe list.

**250 cohort**	
204602_at	DKK1
210512_s_at	VEGFA
211527_x_at	VEGFA
210513_s_at	VEGFA
208570_at	WNT1
213943_at	TWIST1
219480_at	SNAI1
202935_s_at	SOX9
202936_s_at	SOX9
227938_s_at	DLL1
224215_s_at	DLL1
204901_at	BTRC
222374_at	BTRC
1563620_at	BTRC
224471_s_at	BTRC
216091_s_at	BTRC
201566_x_at	ID2
201565_s_at	ID2
221557_s_at	LEF1
221558_s_at	LEF1
210948_s_at	LEF1
205255_x_at	TCF7
205254_x_at	TCF7
244089_at	MYC
202431_s_at	MYC
231183_s_at	JAG1
209097_s_at	JAG1
216268_s_at	JAG1
209099_x_at	JAG1
209098_s_at	JAG1
203753_at	TCF4
222146_s_at	TCF4
213891_s_at	TCF4
212386_at	TCF4
212387_at	TCF4
212382_at	TCF4
228837_at	TCF4
212385_at	TCF4
214702_at	FN1
1558199_at	FN1
212464_s_at	FN1
211719_x_at	FN1
