# Supplementary material for: A SMAD4‐modulated gene profile predicts disease‐free survival in stage II and III colorectal cancer
Source: Cancer Rep (Hoboken). 2021 Jun 10;5(1):e1423. doi: 10.1002/cnr2.1423 (PMC8789617; doi:10.1002/cnr2.1423)
Supplement: Supplementary file 7 — Table S6. Centroid values. [file CNR2-5-e1423-s002.pdf]

Table S6: Centroid values.

	High Risk	Low Risk
204602_at	4.38536437	3.79890292
210512_s_at	10.1615648	10.1596031
211527_x_at	7.5565327	7.57063616
210513_s_at	7.23022614	7.27508986
208570_at	3.66854935	3.77136739
213943_at	5.58459269	4.3051671
219480_at	5.2947878	5.1631849
202935_s_at	10.4631275	10.4795876
202936_s_at	9.78134218	9.83666034
227938_s_at	4.62252401	4.68859476
224215_s_at	6.63703683	6.40974225
204901_at	5.30585387	5.24964914
222374_at	4.33410015	4.4261338
1563620_at	2.42538053	2.42969778
224471_s_at	5.85030821	5.82979228
216091_s_at	4.86021199	4.84661067
201566_x_at	8.05836133	8.13290438
201565_s_at	9.661076	9.68614068
221557_s_at	4.71110761	4.78230487
221558_s_at	7.79007069	7.02437011
210948_s_at	3.968371	3.79579444
205255_x_at	9.01120136	9.19628712
205254_x_at	5.09848662	5.3372572
244089_at	3.81098319	3.89201528
202431_s_at	10.8435205	11.1457131
231183_s_at	6.3086373	5.84496097
209097_s_at	4.20905322	4.25866955
216268_s_at	10.0343182	9.5763767
209099_x_at	10.2490169	9.80134521
209098_s_at	6.64651837	6.16255204
203753_at	8.85288819	7.94596185
222146_s_at	7.51588097	6.63168852
213891_s_at	8.77529588	7.74888632
212386_at	9.17463485	8.13678263
212387_at	7.87042732	6.88318617
212382_at	6.75585635	5.86735866
228837_at	4.31125607	3.76341682
212385_at	6.80337652	5.89531248
214702_at	3.82874475	3.16272535
1558199_at	3.71639723	3.38456923
212464_s_at	11.9033864	10.1668389
211719_x_at	12.0838685	10.4399378
